# HEALing in New Orleans

**Published:** 2006-10

**Authors:** David A. Schwartz, William J. Martin

**Affiliations:** Director, NIEHS and NTP, E-mail: david.schwartz@niehs.nih.gov; Director, Office of Translational Research, E-mail: wjmartin@niehs.nih.gov

A year ago Hurricane Katrina tore through the Gulf Coast states of Louisiana and Mississippi, leaving devastation in its wake—whole communities ripped from their foundations, the displacement of thousands of people from their homes, and a flood of contamination and potential health hazards to be faced. As part of environmental health teams that responded to the disaster, each of us was able to witness firsthand the aftermath of the storm, sights that were both horrendous and deeply compelling to action. A year later, we are excited to announce a new research study that may provide a way for a storm that took so much from so many to give something back. The Head-off Environmental Asthma in Louisiana (HEAL) project will assess the impact on asthma in New Orleans children of environmental health conditions that were caused and exacerbated by Hurricane Katrina, as well as implement an intervention program to address these problems.

Asthma is the most common chronic disease among children in the United States, and its prevalence has been increasing dramatically, particularly among minority children in inner-city urban areas; as much as 24% of such children suffer the disease. Research suggests that these increases may be related to environmental exposures. Inner-city environments typically include high levels of allergens and toxins released from bacteria and mold in the home, high exposures to indoor and outdoor air pollution, environmental tobacco smoke, and stressful living conditions, all of which contribute to asthma’s toll. In addition, the continuity of care and consistent management of a chronic disease such as asthma is often disrupted in inner-city environments by poor access to health care. Indeed, such was the situation in much of New Orleans prior to Hurricane Katrina, whose impact has since served to greatly perturb the environment of the city even more, disrupt its health care delivery system, and further complicate the lives of its children, particularly those with asthma.

The HEAL project, being developed and funded with additional support from the National Center on Minority Health and Health Disparities, is a multi-component, longitudinal study with three main goals: to assess the nature of the environmental and psychosocial impacts of Hurricane Katrina and subsequent flooding on children in New Orleans; to examine the genetic and environmental risk factors for asthma, including genetic susceptibility to mold toxins as well as gene–environment interactions; and to design, implement, and evaluate a case management program to meet the health care needs of children with asthma in a disrupted and highly challenging environment. The third component of the HEAL project will utilize the interventions of the National Cooperative Inner-City Asthma Study.

A team of investigators from Tulane University, the New Orleans Department of Public Health, and clinical research management company Rho, Inc., plan to enroll more than 400 school-aged children, who will be identified by school nurses and surveys. Asthma counselors will work with the family of each child to assess the child’s home and school environment and to educate them in ways to better manage their child’s disease to improve their health. A subset of children will be provided with tools such as HEPA filters for their homes to decrease the presence of allergens and molds. Researchers will assess the effects of the interventions on children’s asthma, as measured by symptom-free days and other end points.

A steering committee aided by both scientific and community advisory groups will oversee the project, which we anticipate will extend over the next 30 months. An important component of the project will be a clear plan for informing the New Orleans community about the goals, implementation, and outcomes of the study. To this end, a community advisory group will represent diverse community perspectives. This group will provide a conduit for community members to present and discuss their needs and concerns related to the HEAL project, as well as their preferences for dissemination of study results to the participants and the community at large. HEAL investigators recognize that sensitivity and responsiveness to these issues will be absolutely vital to the success of this project.

As the residents of New Orleans rebuild their city, the next complex environmental health crisis may be emerging elsewhere in the world. Out of the tragic fate of New Orleans arises an opportunity to learn about exposures and disease that may one day lead to understanding and interventions that will protect children’s health, not only in that future emergency, but every day. It is our hope that contributing to success in this endeavor will be one more way of helping Katrina’s victims to heal.

## Figures and Tables

**Figure:**
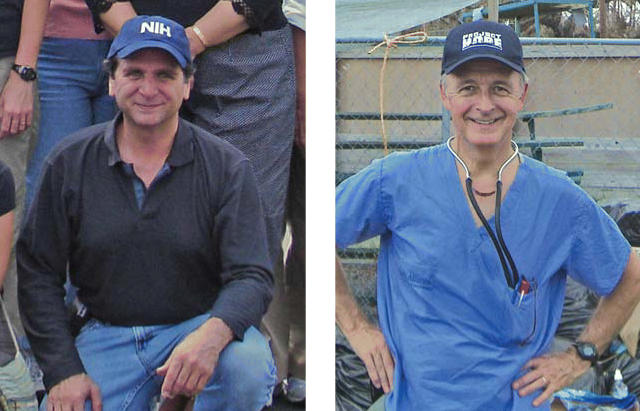
(l–r) David Schwartz with an NIH team in Meridian, Mississippi; Bill Martin with Project Hope in Bay St. Louis, Mississippi.

